# ALLERGIC CONTACT DERMATITIS MIMICKING MAMMARY PAGET'S DISEASE

**DOI:** 10.4103/0019-5154.43210

**Published:** 2008

**Authors:** Kaliyadan Feroze, Jayasree Manoj, S Venkitakrishnan

**Affiliations:** *From the Department of Dermatology, Amrita Institute of Medical Sciences and Research Centre, Kochi, Kerala, India*

**Keywords:** *Allergic contact dermatitis*, *patch testing*, *topical steroids*, *Paget's disease*

## Abstract

A 55-year-old female patient presented to our outpatient department with complaints of persistent erythema, oozing and crusting restricted to the left breast over the last 6 months. The patient underwent investigations to rule out the possibility of mammary Paget'S disease, all of which were negative. A possibility of contact dermatitis to topical medication was considered and confirmed by patch testing.

## Introduction

Contact dermatitis of the breast area can be due to a number of factors including topical applications of various types.^1^–^3^ the lesions present as eczematous dermatitis over the breasts. The closest differential diagnoses are atopic dermatitis and paget's disease of the breasts.

## Case Report

**Fig. 1:**
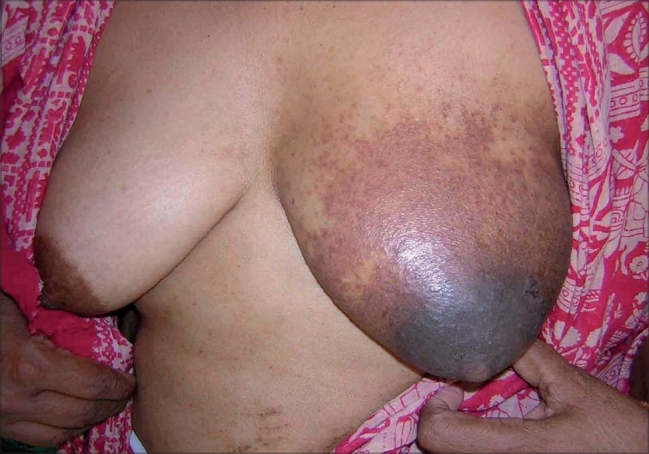
Lesion restricted to the left breast showing erythema with oozing, crusting and scaling

**Fig. 2:**
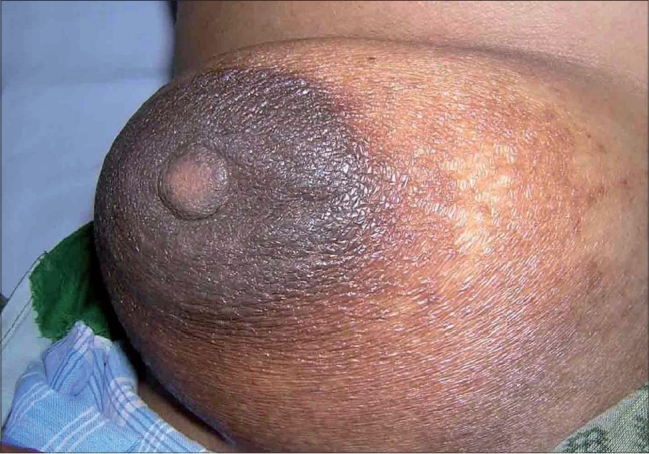
Lesion after 2 weeks of stopping all topical applications

A 55-year-old lady, a college professor, presented to us with a history of persistent itching, erythema, oozing and crusting over the nipple, areola and periareolar region on the left breast of more than 6 months duration. The lesions had been localized to the nipple and immediate periareolar region initially but had started extending recently. She had been using various topical medications for the same including steroids of varying potency. The patient had good response to topical steroids initially but on further use the lesion appeared to be getting worse. The patient had no other significant co-morbidity and was in otherwise good health. On examination we found an ill-defined area of scaling, erythema and minimal crusting over the over the nipple, areola and peri-areolar region on the left breast (Fig. 1). There was minimal induration over the nipple and immediate peri-areolar area and no significant tenderness or bleeding on touch. Patient had no significant lymphadenopathy. The unilateral nature of the central lesions and the apparent lack of response to topical steroids made us consider a possibility of mammary Paget's disease, for which the patient was evaluated. Routine blood and urine investigation were within normal limits. Potassium hydroxide smears and cytology findings were normal. Mammography showed no evidence of any micro calcification or significant breast tissue involvement suggestive of malignancy. A skin biopsy was taken form two sites and both the specimens showed features of a dermatitis with spongiosis and moderate lymphocytic infiltration. Based on the investigation results we ruled out the possibility of Paget's disease and considered an alternative possibility of a primary non-specific dermatitis with a persistent contact dermatitis to topical applications. We initially patch tested the patient with the commercial products she was using, including brands of fluticasone and betamethasone. Interestingly all the products showed a strong positive reaction after 48h. We then patch-tested the patient with multiple antigens including topical antibiotics, preservatives and bases. The patch tests showed a strongly positive reaction to propylene glycol, parabens and chlorocresol and a moderate reaction to both neomycin and gentamycin. We also carried out an intradermal test with hydrocortisone and betamethasone solutions in saline, which gave a negative result. Based on the patch test findings we asked the patient to stop all topical preparations and started the patient on saline compresses and plain Vaseline along with a short course of systemic steroids. The lesions improved and by the end of two weeks had subsided almost entirely with only a mild residual hyperpigmentation (Fig. 2).

## Discussion

In our case the unilateral presentation and the apparent lack of response to topical steroids made us think on the lines of mammary Paget's. Most eczematous lesions of the breast tend to be bilateral like seborrheic and atopic dermatitis though there have been reports of chronic unilateral dermatitis of the nipple areolar complex.^4^ The diagnosis of mammary Paget's was excluded in our case based on the cytology, mammography and histopathology findings. The history of apparent worsening of the lesions after an initial improvement made us think of a possibility of an allergic contact dermatitis to the topical applications used by the patient. Ointment bases and vehicles are known to cause various kinds of contact allergies, prominent among them being those due to chlorocresols, parabens and propylene glycol.^5^,^6^ Topical corticosteroids per se can also cause an allergic contact dermatitis, especially tixocortol pivalate and budesonide and need to be tested in suspected cases, either as a patch test in an ethanol base or as an intradermal test in saline.^7^–^9^ Nonhalogenated topical steroids are considered to be more frequent sensitizers than halogenated molecules.^7^
